# SARS-CoV-2 Infection in Cancer Patients: A Picture of an Italian *Onco-Covid* Unit

**DOI:** 10.3389/fonc.2020.01722

**Published:** 2020-08-19

**Authors:** Maria Lucia Reale, Paolo Bironzo, Valentina Bertaglia, Erica Palesandro, Gianmarco Leone, Fabrizio Tabbò, Maristella Bungaro, Marco Audisio, Annapaola Mariniello, Simonetta G. Rapetti, Rosario F. Di Stefano, Elisa Artusio, Enrica Capelletto, Paola Sperone, Adriana Boccuzzi, Marco Calandri, Alberto Perboni, Umberto Malapelle, Francesco Passiglia, Silvia Novello

**Affiliations:** ^1^Department of Oncology, University of Turin, San Luigi Gonzaga Hospital, Turin, Italy; ^2^Emergency Medicine, San Luigi Gonzaga Hospital, Turin, Italy; ^3^Radiology Unit, Department of Oncology, San Luigi Gonzaga Hospital, University of Turin, Turin, Italy; ^4^Pneumologia, San Luigi Gonzaga Hospital, Turin, Italy; ^5^Department of Public Health, University of Naples Federico II, Naples, Italy

**Keywords:** COVID-19, lung cancer, cancer patients, asymptomatic patients, Italian retrospective study

## Abstract

**Background:** The world, and Italy on the front lines, has experienced a major medical emergency due to the novel coronavirus outbreak. Cancer patients are one of the potentially most vulnerable cohorts of people, but data about their management are still few.

**Patients and Methods:** In this monocentric retrospective study we included all SARS-CoV-2 oncological patients accepted, between March 27th and April 19th 2020, at the Onco-COVID Unit at San Luigi Gonzaga Hospital, one of the few Italian oncological-COVID wards. Data were obtained from medical records.

**Results:** Eighteen cancer patients with COVID-19 were included. The mean (±SD) age of patients was 67 ± 14 years, 89% were men. Seven (39%) developed infection in communities and 11 (61%) during hospitalization. Lung cancer was the most frequent type of cancer (10, 56%). Seven patients (39%) were symptomatic for COVID-19 at the time of diagnosis and symptoms began 2 (±2) days before. The most common were shortness of breath and diarrhea. Fever was present in 5 patients (28%). Among the 11 asymptomatic patients, 8 (73%) became symptomatic during the hospitalization (mean time of symptoms onset 4 days ±4). Six patients (33%) were on active anti-tumor treatment: 2 (33%) received anti-tumor therapy within 2 weeks before the infection diagnosis and 2 (33%) continued oncological treatment after SARS-CoV-2 positivity. Eight (44%) patients died within a mean of 12 days (±8) from the infection diagnosis.

**Conclusions:** Our series confirms the high mortality among cancer patients with COVID-19. The presence of asymptomatic cases evidences that typical symptoms and fever are not the only parameters to suspect the infection. The Onco-Covid unit suggests the importance of a tailored and holistic approach, even in this difficult situation.

## Introduction

Over the past few months, the world has to face a major medical emergency due to the novel coronavirus SARS-CoV-2 outbreak. The high transmissibility rate and the rapid spread of the virus have led the World Health Organization (WHO) to declare the state of pandemic on March 11th 2020. More than 6 millions of cases and 370,000 deaths from COVID-19 (the disease caused by SARS-CoV-2) have been confirmed worldwide and Italy rapidly became one of the most affected countries (1, 2).

Given the immunosuppression caused by the disease itself and by antitumor treatments, cancer patients seem to be a vulnerable population. National and international scientific societies have drawn up specific recommendations in order to establish the priorities in the oncological treatment and to mitigate the negative effects of the pandemic on the management of cancer patients (3–5).

Liang et al. reported a higher risk of developing the infection, of critical events and of mortality rates for 18 Chinese cancer patients compared to the general population (6). Another retrospective study conducted in three hospitals in Wuhan confirmed high rates of severe events—exacerbated by the administrations of anticancer treatment within 14 days of infection—and of mortality (7). High rates of 30-day all-cause mortality has also been confirmed by Cancer Consortium (CCC19) data analysis (8). Myashita et al. stratifying 334 American cancer patients with COVID-19 infection by age groups, detected a higher mortality in patients younger than 50 years (9).

More data are needed, however, to better understand the risk and the best management of COVID-19 infection in cancer patients. Issues concerning the prevalence of asymptomatic patients, the time of symptoms onset, the best therapeutic approach (antiviral and oncological), and the infection evolution are still unsolved. Since the resolution of the current health crisis looks as a long and not easy process, the collection of real-world data is a valuable strategy. This retrospective study aims to collect epidemiological, clinical, laboratory, and therapeutic data from SARS-CoV-2 positive cancer patients hospitalized at the Onco-Covid unit in San Luigi Gonzaga Hospital, Italy, one of the few oncological wards for cancer patients with SARS-Cov-2 infection, in order to provide a deeper insight into the clinical evolution of infection in cancer patients, particularly in lung cancer patients.

## Methods

### Study Population, Setting, and Data Collection

The study was approved by the Ethics Committee of the AOU San Luigi Gonzaga, Orbassano (Turin). The requirement for specific informed patient consent was waived due to the emergency status, upon the approval of the Ethics Committee. However, patients provided a general written informed consent for clinical data collection at the time of hospitalization.

This retrospective study included all SARS-CoV-2 oncological patients accepted at the Onco-Covid Unit at San Luigi Gonzaga Hospital, Orbassano, between March 27th and April 19th 2020.

Clinical data was retrospectively retrieved from the medical records, including demographic and clinical features, laboratory, and radiological findings at presentation and during the hospitalization. In particular, following internal hospital protocol, a chest-X-ray or a chest ultrasound was executed at baseline. In case of severe respiratory illness in patients with a not poor prognosis, a computed tomography (CT) scan was also obtained. The radiological assessment was monitored during the hospitalization. However, all laboratory and radiologic tests were performed at the discretion of the treating physicians, taking into consideration patients' general conditions and prognosis.

Patient data were censored at the time of data cutoff, which coincides with the closing date of the ward and occurred on April 19th, 2020.

A confirmed case of Covid-19 was defined by a positive result on a reverse-transcriptase–polymerase-chain-reaction (RT-PCR) assay (VIASURE SARS-CoV-2 Real Time PCR Detection Kit, targeting the SARS-CoV-2 S gene) performed on a nasopharyngeal swab- derived specimen, following the WHO criteria (10).

The ratio of arterial oxygen partial pressure (PaO_2_ in mmHg) to fractional inspired oxygen (FiO_2_), defined as P/F, was used as clinical indicator of hypoxemia.

Disease response (investigator-assessed) was defined according to Response Evaluation Criteria In Solid Tumors (RECIST) v. 1.1 criteria and to clinical evaluation.

The terms “best supportive care” were used to define care given to patients with no more indications to active antitumor treatment, aiming at improve the quality of life by preventing or treating symptoms.

### Statistical Analysis

Descriptive statistics were used to summarize the data; results were reported as means and standard deviations (SD). Categorical variables were summarized as counts and percentages. Analysis was performed with IBM SPSS software.

## Results

### Clinical Characteristics of the Patients

From March 27th to April 19th 2020, we identified 18 cancer patients with confirmed SARS-CoV-2 positivity at the Onco-Covid Unit.

The demographic and clinical characteristics are shown in Table 1. The mean (±SD) age of the patients was 67 ± 14 years (range 26–84); 16 (89%) were men. Twelve patients (66%) were current or former smokers.

**Table 1:** Clinical characteristics of the patients at baseline^+^.

**Clinical characteristics of the patients at baseline**	
**Number of patients**	18
**Age (range)**	Mean 67 years ± 14 (26–84)
**Sex—no. (%)**	Male: 16 (89)
	Female: 2 (11)
**Smoking habit—no. (%)**	Never: 1 (6)
	Former: 6 (33)
	Current: 6 (33)
	Missing data: 5 (28)
BMI^*^	Mean: 24 ± 4 (16–30)
**Coexisting disorder—no. (%)**	
None	6 (33)
Chronic obstructive pulmonary disease	2 (11)
Diabetes mellitus	2 (11)
Hypertension	9 (50)
Ischemic Cardiomyopathy	1 (6)
**Concomitant medications—no. (%)**	
Insulin/metformin	2 (11)
Antihypertensive	7 (39)
Anticoagulant/antiplatelet	8 (44)
**ECOG PS—no. (%)**	
ECOG PS 0	2/18 (11)
ECOG PS 1	5/18 (28)
ECOG PS 2	9/18 (50)
ECOG PS 3	2/18 (11)
ECOG PS 4	0/18 (0)
**Steroid treatment—no. (%)**	4 (22)
**Oxygen treatment—no. (%)**	0 (0)

+ *Plus-minus values are means ± SD. Percentages may not total 100 because of rounding*.

* *The body-mass index in the weight in kilograms divided by the square of the height in meters. Data on body-mass index were missing for 5 patients*.

Chronic medical conditions, beside the oncological disease, were present in 12 (67%) patients. Nine patients (50%) had hypertension, 2 (11%) chronic obstructive pulmonary disease, 2 (11%) diabetes mellitus, and 1 (6%) ischemic cardiomyopathy. Seven patients (39%) were in antihypertensive treatment, 8 (44%) in anticoagulant/antiplatelet therapy for primary or secondary prevention, 2 (11%) in hypoglycemic therapy. Before the diagnosis of infection, 4 patients (22%) received systemic glucocorticoids and none of them was under oxygen therapy.

Most patients presented with an Eastern Cooperative Oncological Group (ECOG) Performance Status (PS) of 2 (9, 50%) and 1 (5, 28%).

As shown in Table 2, lung cancer was the most frequent type of cancer (10, 56%), followed by blood/bone marrow cancer (3, 17%). Fourteen patients (78%) were diagnosed with stage IV, with 11 (61%) patients reporting a lung/pleura involvement. In most of cases (13, 72%), the diagnosis of cancer had occurred in the last 12 months.

**Table 2:** Cancer type and treatment history.

Cancer type and treatment history^*^	
**Primary cancer site no. (%)**	
Lung	10/18 (56)
Colon	1/18 (6)
Pancreas	1/18 (6)
Bladder	1/18 (6)
Blood/bone marrow	3/18 (17)
Brain (low grade)	1/18 (6)
Neuroendocrine cells (Paraganglioma)	1/18 (6)
**Stage IV—no. (%)**	14/18 (78)
**Presence of lung/pleura disease—no. (%)**	11/18 (61)
**Time from oncological diagnosis to COVID-19 positivity—no. (%)**	
≥24 months	2/18 (11)
24–12 months	3/18 (17)
<12 months	13/18 (72)
**Oncological indications—no. (%)**	
Active anti-tumor treatment	6/18 (33)
New diagnosis-active treatment candidates	2/18 (11)
Best supportive care	10/18 (56)
**Line of therapy for patients in active anti-tumor treatment—no. (%)**	
Neoadjuvant	0/6 (0)
Adjuvant	0/6 (0)
I line	4/6 (67)
II line	1/6 (17)
III line and beyond	1/6 (17)
**Type of therapy for patients in active anti-tumor treatment—no. (%)**	
Chemotherapy	2/6 (33)
Immunotherapy	3/6 (50)
Tyrosine Kinase Inhibitors	1/6 (17)
**Time from last anticancer drug administration to COVID-19 positivity for patients in active anti-tumor treatment—no. (%)**	
3 weeks	2/6 (33)
2 weeks	1/6 (17)
1 week	1/6 (17)
Treatment ongoing (before and after the infection)	2/6 (33)

* *Percentages may not total 100 because of rounding*.

Ten patients (56%) had already received a clinical indication to home-based best supportive care before the infection; six patients (33%) were, on the contrary, on active anti-tumor treatment, a first line therapy in 4 (67%) cases. Two patients (11%), with a new cancer diagnosis were potentially candidates to medical treatment, but were still naïve at the time of infection. The last anti-cancer therapy received by patients on treatment was chemotherapy, immunotherapy or tyrosine kinase inhibitors (TKIs) in 2 (33%), 3 (50%), and 1 (17%) patients, respectively. Two patients (33%) received anti-tumor therapy within 3 weeks before SARS-CoV-2 positivity and other two (33%) within 2 weeks. Two patients (33%) continued oncological treatment (TKIs and chemotherapy) after the infection diagnosis.

### Clinical, Laboratory, and Radiological Features of the Patients at Baseline (First SARS-CoV-2 Positivity)

The clinical, laboratory, and radiologic findings of the patients at SARS-CoV-2 infection diagnosis were summarized in Table 3.

**Table 3:** Clinical, laboratory, and radiological features of the patients at baseline (First SARS-CoV-2 positivity).

Clinical, laboratory, and radiological features of the Patients at Baseline (First SARS-CoV-2 Positivity)^*^	
**Source of infection—no. (%)**	
Community	7/18 (39)
Nosocomial transmission	11/18 (61)
**Symptomatic patients—no. (%)**	7/18 (39)
**Mean duration of symptoms before diagnosis of infection—days (range)**	2 ± 2 (0–7)
**Symptoms—no. (%)**	
Shortness of breath	2/18 (11)
Cough	1/18 (6)
Sputum production	1/18 (6)
Sore throat	1/18 (6)
Diarrhea	2/18 (11)
Fatigue	1/18 (6)
**Temperature—n./total no. (%)**	
<37.5°C	13/18 (72)
37.5–38°C	1/18 (6)
38.1–39°C	4/18 (22)
>39°C	0/18 (0)
**Peripheral oxygen saturation (SpO**_**2**_**) n./total no. (%)**	
>95%	7/18 (39)
91–95%	8/18 (44)
85–90%	0/18 (0)
<85%	3/18 (17)
**P/F no./total no. (%)**	
<200	3/18 (17)
200–300	7/18 (39)
300–400	1/18 (6)
>400	2/18 (11)
Unknown	5/18 (28)
**Distribution of laboratory findings no./total no. (%)**	
Lymphocyte count decrease	5/18 (28)
Platelet count decrease	4/18 (22)
C-reactive protein ≥5 mg/dl	14/18 (78)
Procalcitonin ≥0.5 ng/ml	1/18 (6)
Lactate dehydrogenase ≥240 U/liter	11/18 (61)
**Chest radiography findings—no./total no. (%)**	
Bilateral infiltrates	5/12 (42)
Parenchymal consolidation	2/12 (17)
Clear	5/12 (42)
**Abnormalities on chest ultrasound—no./total no. (%)**	
Negative	1/5 (20)
B-lines	3/5 (60)
Subpleural consolidations, irregular pleural line and B-lines	1/5 (20)
**Abnormalities on chest CT—no./total no. (%)**	
Bilateral ground-glass opacities	2/3 (67)
Parenchymal consolidation	1/3 (33)
Clear	0/3 (0)

* *Plus-minus values are means ± SD. Percentages may not total 100 because of rounding*.

There were two main clusters of patients: 7 (39%) patients who developed infection in their communities and 11 (61%) during the hospitalization (reasons of hospitalization are reported in Supplementary Table 1.)

Seven patients (39%) were symptomatic for COVID-19 infection at the time of nasopharyngeal swab. The mean duration of symptoms before the diagnosis was 2 ± 2 days. The most common were shortness of breath and diarrhea both occurring in 2 patients (11%). Among the 11 asymptomatic patients, 8 (72%) became symptomatic during the hospitalization (mean time of symptoms onset: 4 days ±4). Documented fever was present in 5 patients (28%).

Peripheral oxygen saturation (SpO_2_) overcame the 90% in 15 patients (83%) and was <85% in 3 patients (17%). P/F was <200 in 3 patients (17%), between 200 and 300 in 7 (39%) patients and higher than 300 in 3 (17%).

C-reactive protein increase (≥5 mg/dl) was detected in 14 (78%) patients. High lactate dehydrogenase levels (≥240 U/l) were present in 11 patients (61%), while lymphocytopenia and thrombocytopenia in 5 and 4 (28 and 22%), respectively. A procalcitonin increase (≥0.5 ng/ml) was detected only in 1 patient (6%).

A chest radiograph was obtained in 12 patients (67%). The radiographs showed bilateral infiltrates and parenchymal consolidation in 5 and 2 cases (42 and 17%), respectively. A thoracic ultrasound was executed in 5 patients: no anomalies in one case (20%), B-lines in 3 patients (60%), B-lines plus sub pleural consolidations plus irregular pleural line in another case (20%). In 5 patients (42%) no radiological anomalies were evident. A computed tomography (CT) scan of the chest was obtained in 3 patients (17%), with 2 scans showing newly- appeared bilateral ground glass opacities in one case and parenchymal consolidations in the other one.

### Treatment, Clinical Evolution and Outcomes

**Figure 1:**
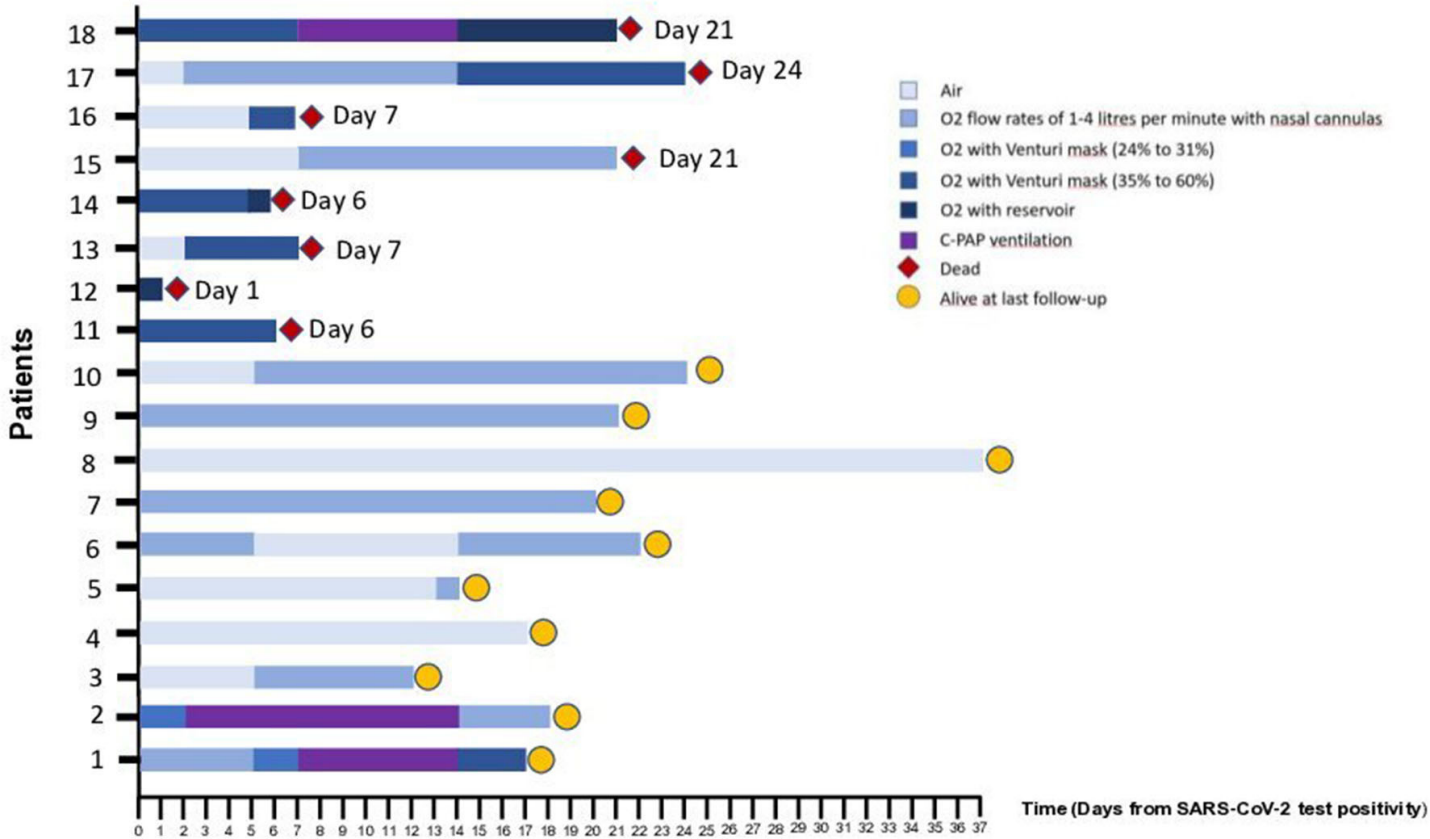
Oxygen therapy for Individual Patients Included in the Case Series. As of April 19th, 2020, a total of 8 patients (44%) had died. Five patients (28%) had been discharged from the hospital. Here we report the oxygen therapy received by each patient during the hospitalization. The number of each patient corresponds to that reported in Supplementary Table 2 showing individual clinical characteristics and outcomes.

The trend of the P/F analyzed after 7 and 14 days (±2) after the SARS-CoV-2 positivity is shown in Table 4: P/F was <300 in 3 (21%) and four (30%) patients at the two reported intervals, respectively. Sixteen patients (89%) received oxygen therapy with nasal cannula (1–4 L/min), Venturi mask (24–31% or 35–60%), reservoir or non-invasive ventilation with continuous positive airway pressure (CPAP), as reported in Figure 1. None of them received endotracheal intubation or invasive ventilation.

**Table 4:** Treatment, clinical evolution and outcomes^*^.

**P/F after 7 ± 2 days n./total no. (%)**	
<200	1/14 (7)
200–300	2/14 (14)
300–400	4/14 (29)
>400	1/14 (7)
Unknown	6/14 (43)
**P/F after 14 ± 2 days n./total no. (%)**	
<200	2/13 (15)
200–300	2/13 (15)
300–400	4/13 (31)
>400	1/13 (8)
Unknown	4/13 (31)
**Treatments no. (%)**	
Hydroxychloroquine	7/18 (39)
Darunavir/ritonavir	4/18 (22)
Intravenous antibiotics	15/18 (83)
Systemic glucocorticoids	14/18 (78)
**Distribution of laboratory findings n./total no. (%)**	
Lymphocyte count decrease	7/18 (39)
Platelet count decrease	6/18 (33)
C-reactive protein ≥5 mg/liter	12/18 (67)
Procalcitonin ≥0.5 ng/ml	1/18 (6)
Lactate dehydrogenase ≥240 U/liter	8/18 (44)
**Control chest radiography findings—n./total no. (%)**	
Improvement	2/12 (17)
Unchanged appearance	2/12 (17)
Deterioration	3/12 (25)
Not executed	5/12 (42)
**Mean length of hospital stay from hospitalization at data cut-off—days (range)**	30 ± 14 (0–53)
**Mean length of hospital stay from COVID19 positivity at data cut-off—days (range)**	16 ± 9 (0–37)
**Nasal swab—n./total no. (%)**	
Negative	6/18 (33)
Positive	4/18 (22)
Not done	8/18 (44)
**Complications no. (%)**	
Septic shock	1/18 (6)
Acute respiratory distress syndrome	3/18 (17)
Progression of disease	9/18 (50)
None	4/18 (22)
**Clinical outcomes at data cutoff—no. (%)**	
Hospitalization	4/18 (22)
Admission to a mild intensive care COVID department	1/18 (6)
Discharge from hospital (active treatment)	1/18 (6)
Discharge from hospital (best supportive care)	4/18 (22)
Death	8/18 (44)

* *Plus-minus values are means ± SD. Percentages may not total 100 because of rounding*.

Laboratory findings during the hospitalization are reported in Table 4.

A second chest X-ray was performed after 14 days from the diagnosis of infection in 7 patients (58%), showing a radiological improvement in 2 (17%), deterioration in 3 (25%), and stability in 2 (17%) patients. In one of the latter, affected by lung cancer and treated with a combination of TKI and COVID-19 directed therapy, a CT scan was also performed, showing both the persistence of inflammatory lung infiltrates and a tumor partial response.

In absence of robust evidences therapeutic management was based on local guidelines and daily multidisciplinary evaluations involving oncologists together with pulmonologists, intensive care, and infectious disease specialists. Seven (39%) patients were prescribed hydroxychloroquine for up to 10 days and 4 (22%) antiviral therapy with lopinavir/ritonavir or darunavir/ritonavir for 7 consecutive days. Antibiotics and systemic glucocorticoids treatment were administered to 15 (83%) and 14 patients (78%), respectively, and just 4 of them were in on steroid treatment before the diagnosis of infection.

The most common complication was the progression of oncological disease (9, 50%) followed by Acute Respiratory Distress Syndrome (ARDS) (3, 17%) and septic shock (1, 6%). Nasal and/or pharyngeal swabs tested for SARS-CoV-2 were collected after 14 days from the first positivity in 10 (56%) patients: 6 (33%) resulted negative (with a second confirmatory test performed at 24 h from the previous negative one) and 4 (22%) persistently positive.

As of April 19th, 8 (44%) died with a mean of 12 days (±8, range 0–24) from the diagnosis of COVID-19 infection to death. Five (28%) patients have been discharged from the Onco-Covid Unit but remained in the hospital, 4 (22%) have been transferred to another low intensive care COVID department and 1 (6%) to a mild intensive care unit. Five patients (28%) have been discharged from the hospital, 4 (22%) of them with the indication to best supportive care and 1 (6%) to active antitumor treatment. The mean length of hospitalization at data cut-off was 30 days ±14 (0–53), while it resulted 16 ± 9 days (0–37) when calculated from COVID 19 positivity (characteristics and outcomes of individual patients included in the analysis are reported in Supplementary Table 2).

## Discussion

This monocentric retrospective case series describes the 18 cancer patients, whose 10 (56%) affected by lung cancer, who developed laboratory-confirmed COVID-19 infection and have been hospitalized at the Onco-Covid Unit of San Luigi Gonzaga Hospital, one of the few oncological wards dedicated to cancer patients with SARS-Cov-2 infection, between March 27th and April 19th, 2020. In our series, 11 (61%) patients developed infection during their hospitalization at our oncology department. The internal transmission was probably due to a patient's contact with a caregiver subsequently known to have been ill, only few days after the identification of the first case in our Region. When the first evidence of infection occurred, we performed screening test even in asymptomatic hospitalized patients in order to provide a rapid isolation of any positive case. Out of 24 hospitalized patients, 10 resulted positive for SARS-CoV-2 infection. Thus, after the transfer of negative patients to other wards, we created the Onco-Covid Unit. From that moment we admitted only infected cancer patients coming from the Emergency Department, where nasopharyngeal swab was always performed before hospitalization. We also empowered the use of personal protective equipment (PPE) such as hand sanitizer, isolation gown, filtered masks, face shield or goggles, and gloves. In their analysis, Zhang et al. reported that 29% of patients had developed COVID-19 infection during hospitalization (7). In a Chinese retrospective study among the 138 hospitalized patients included, 41% were presumed to have been infected in hospital−5 of them coming from the oncology department—and 29% were health care workers (11). These data underline the importance of both control measures and PPE adoption to slow down the infection risk. In our center, we regularly perform a triage of all subjects before entering the hospital with body temperature measure and questionnaire about epidemiological status and clinical conditions. We also forbid hospital access to caregivers, even after the first epidemic phase, although we acknowledge its heavy emotional impact on hospitalized patients experiencing complete isolation. Caregivers' visits are allowed in case of rapid clinical deterioration with the proper use of PPE.

The screening testing executed in the oncology ward, provided the opportunity to observe the clinical evolution of asymptomatic SARS-CoV-2 infection in the oncological population. Indeed, only seven 7 patients (39%) were symptomatic, a lower percentage as compared to what has been previously reported (7). The mean time to the onset of any infection-related symptoms in asymptomatic population was 4 days, with 3 patients who remained asymptomatic during the entire observation period. To our knowledge, this is the first reported series describing the evolution of infection of SARS-CoV-2 in asymptomatic cancer patients.

In symptomatic patients, shortness of breath and diarrhea were the most common presenting symptoms (11%), with a mean duration before the diagnosis of infection of 2 days (0–7 days). Only 5 patients (28%) had fever and 3 (17%) patients showed a <85% peripheral oxygen saturation at the time of infection diagnosis. Altogether, these data suggest that such symptoms and fever may not be the only useful parameters to suspect COVID-19 in cancer patients. Lung cancer, as in other analyses, was the most frequent type of cancer (56%) and most patients (78%) were diagnosed with stage IV disease (6, 7). Furthermore, data from the TERAVOLT registry suggests high mortality and low admission to intensive care in patients with thoracic cancer (12).

The majority of patients had chronic illnesses beside the oncological disease, most commonly hypertension. C-reactive protein increase and high lactate dehydrogenase levels were frequent laboratory alterations at baseline.

Radiological patterns of COVID-19 lung disease were extremely heterogeneous showing bilateral infiltrates/parenchymal consolidations or a clear appearance (42%).

Regarding antiviral interventions, 39% received off-label hydroxychloroquine and 22% lopinavir/ritonavir (or darunavir/ritonavir) following local guidelines subsequently modified because of hydroxychloroquine suspension by regulatory agencies (13, 14). We have insufficient information, however, to report associated outcomes. The absence of patients treated with immune suppressive drugs and with remdesivir, despite their promising results, is due to the high rate of patients with a poor performance status as well as to the treatment criteria established by local guidelines (13, 15). Only one patient was deemed, based on respiratory illness and oncological prognosis, to these treatments but, fortunately, his clinical conditions improved with the only combination of hydroxychloroquine and darunavir/ritonavir. None of the patients was intubated, reflecting the high rate of patients with a compromised status and without active antitumor indications even before SARS-CoV-2 infection. The choice not to intubate critical patients was always based on a multidisciplinary discussion tailored on every single case, taking into consideration the patient's clinical condition and prognosis that, for most of them, was unfortunately poor.

Systemic glucocorticoids were administered to 14 patients (78%), mostly taking into account some promising results of glucocorticoids in improving clinical outcomes in patients with respiratory illness, even if data were still conflicting (16, 17). More recently, however, the benefit of glucocorticoids has been demonstrated in the preliminary report of the RECOVERY trial in which the use of dexamethasone resulted in lower mortality among those who were receiving either invasive mechanical ventilation or oxygen (18).

To date, recent anticancer treatment appears to increase the risk of severe events in case of Sars-Cov-2 infection, except for the results of the UK Coronavirus Cancer Monitoring Project where recent chemotherapy use in cancer patients before infection was not significantly associated with increased mortality (6, 7, 19). However, in our case series two cancer patients continued on receiving anti-tumor treatment after having acquired COVID-19 infection during the hospitalization. One of them was a 26-year-old man with a new diagnosis of acute promyelocytic leukemia and the other one was a 46-year-old man with a new diagnosis of EGFR (Epidermal Growth Factor Receptor) mutated non-small cell lung cancer. In the first case the diagnosis of the most curable subtype of acute myeloid leukemia that, however, can have a substantial rate of mortality within the first week/month and the young age, were the cornerstone for deciding to start the specific hematological treatment even in the presence of SARS-CoV-2 infection. The management of patient was based on a daily collaborative work with hematologists. A bone marrow aspirate performed at the end of April showed flow cytometric remission. In the second case, as previously reported, after a careful assessment of risks and benefits, the patient was treated with a combination of osimertinib and COVID-19 directed agents under a close clinical and laboratory monitoring achieving a radiological partial response.

The most common complications in the whole study population were tumor progression (50%), ARDS (17%) and septic shock (6%). The case fatality rate of 44% in this series (to date) is higher than already reported by other Authors (6, 7). One reason might be ascribed to the high percentage of patients already hospitalized because of a clinical deterioration (22%).

For this latter reason, clinical management might have been less aggressive, as prognosis was deemed poor even before the diagnosis of COVID-19. Moreover, our case fatality rate may be underestimated, considering that 4 patients were discharged with the indication to best supportive care at the time data were censored.

Globally, our series confirms a higher mortality rate in cancer patients with COVID-19 when compared to regional mortality rate (8% as of June 1st), even if a direct comparison with the general population was not performed (20). Importantly, however, 50% of patients in our series had an ECOG performance status of 2 at the time of hospital admission, only partially representing a typical cancer patients cohort. Moderate or poor ECOG performance status is a well-known poor prognostic factor, further explaining the high mortality rate observed.

Our study has many important limitations, especially its retrospective nature. For this reason, some cases had either missing laboratory data or radiological evaluations or both (all exams were performed at the discretion of the treating physicians). Second, the sample size is small, limiting its generalizability. Third, as we focused on the entire population of cancer patients, sample is highly heterogeneous regarding tumor type, stage of disease, performance status, treatments. Finally, 5 patients (28%) remained in hospitals at the time of data censoring on April 19th, 2020; as a result, outcomes for those patients were not known.

To our knowledge, this is the first report of a pure Onco-Covid reality. Although our study reinforces the fragility of oncological patients during this emergency, cancer is not a homogeneous disease and potential differences in therapeutic options and prognosis are substantial, even in advanced stage. For this reason, oncologists have to be involved in the management of all cancer patients affected by COVID-19 for a shared decision-making process aimed at tailoring decisions on every patient reducing any risks of under- or over-treatment. The Onco-Covid Unit has represented an example, although perfectible, of this tailored and holistic approach, even in a challenging situation like SARS-CoV-2 pandemic.

## Data Availability Statement

The raw data supporting the conclusions of this article will be made available by the authors, without undue reservation.

## Ethics Statement

This study was approved by the Ethics Committee of the AOU San Luigi Gonzaga, Orbassano (Turin). The requirement for informed patient consent was waived due to the emergency status.

## Author Contributions

MR and PB: study concept and design. MR, VB, and PB: data analysis and interpratation. All authors: data acquisition, manuscript preparation, and review. MR and FP: manuscript editing.

## Conflict of Interest

The authors declare that the research was conducted in the absence of any commercial or financial relationships that could be construed as a potential conflict of interest. The Editorial Office has been notified of the past co-authorships between the reviewers MS, DG, and VS and several of the authors UM, FT, and SN and ensure that the review process met the standards of a fair and objective review. This transparency statement is being included in acknowledgment of the crucial collaborations ensuring the swift dissemination of research in response to the COVID- 19 pandemic.
