# Supplementary material for: SARS-CoV-2 Infection in Cancer Patients: A Picture of an Italian Onco-Covid Unit
Source: Front Oncol. 2020 Aug 19;10:1722. doi: 10.3389/fonc.2020.01722 (PMC7466731; doi:10.3389/fonc.2020.01722)
Supplement: Supplementary file 1 [file Table_1.DOCX]

| **Reasons of hospitalization no. (%)*** |  |
| --- | --- |
| SARS-CoV-2 pneumonia | 5/18 (28) |
| SARS-CoV-2 septic shock | 1/18 (6) |
| SARS-CoV-2 infection in newly diagnosed leukemia | 1/18 (6) |
| Respiratory failure in newly diagnosed lung cancer | 3/18 (17) |
| Severe anemia | 1/18 (6) |
| Clinical deterioration | 4/18 (22) |
| Pleural empyema | 1/18 (6) |
| Severe hyponatremia | 1/18 (6) |
| Pneumonia (other than SARS-CoV-2) | 1/18 (6) |

**Supplementary Table 1: reasons of hospitalization**. *Percentages may not total 100 because of rounding.
