# Supplementary material for: SARS-CoV-2 Infection in Cancer Patients: A Picture of an Italian Onco-Covid Unit
Source: Front Oncol. 2020 Aug 19;10:1722. doi: 10.3389/fonc.2020.01722 (PMC7466731; doi:10.3389/fonc.2020.01722)
Supplement: Supplementary file 2 [file Table_2.DOCX]

| **Patient** | **Sex** | **Age** | **ECOG PS** | **Primary cancer site** | **Symptoms at baseline (first Sars-Cov-2 positivity)** | **Main laboratory findings at baseline** | **Treatment for COVID-19** | **Complications** | **Clinical outcomes at data cutoff** |
| --- | --- | --- | --- | --- | --- | --- | --- | --- | --- |
| Patient 1 | xy | 46 | 1 | Lung | Yes | C-reactive protein ≥5 mg/dl | Yes (hydroxychloroquine + darunavir/ritonavir) | None | Hospitalization |
| Patient 2 | xy | 63 | 0 | Neuroendocrine cells | No | Lymphocytes decreased (G2);  C-reactive protein ≥5 mg/dl;  Procalcitonin ≥0.5 ng/ml;  Lactate dehydrogenase ≥240 U/liter | Yes (hydroxychloroquine) | None | Discharge from hospital (active treatment) |
| Patient 3 | xy | 76 | 1 | Lung | Yes | Lymphocytes decreased (G3);  Platelet count decrease (G1);  C-reactive protein ≥5 mg/dl;  Lactate dehydrogenase ≥240 U/liter | Yes (hydroxychloroquine) | Acute respiratory distress syndrome | Admission to a mild intensive care COVID department |
| Patient 4 | xy | 26 | 0 | Blood/Bone Marrow | Yes | Platelet count decrease (G3);  C-reactive protein ≥5 mg/dl;  Lactate dehydrogenase ≥240 U/liter | Yes (hydroxychloroquine) | None | Hospitalization |
| Patient 5 | xy | 63 | 2 | Pancreas | No | C-reactive protein ≥5 mg/dl;  Lactate dehydrogenase ≥240 U/liter | No | None | Hospitalization |
| Patient 6 | xy | 65 | 2 | Lung | No | C-reactive protein ≥5 mg/dl; | No | Progression of disease | Discharge from hospital (best supportive care) |
| Patient 7 | xy | 65 | 2 | Lung | No | Lymphocytes decreased (G1);  C-reactive protein ≥5 mg/dl;  Lactate dehydrogenase ≥240 U/liter | No | Progression of disease | Discharge from hospital (best supportive care) |
| Patient 8 | xy | 76 | 2 | Brain | No | Lactate dehydrogenase ≥240 U/liter | Yes (hydroxychloroquine and darunavir/ritonavir) | Progression of disease | Discharge from hospital (best supportive care) |
| Patient 9 | xy | 81 | 2 | Lung | No | Lymphocytes decreased (G1);  C-reactive protein ≥5 mg/dl; | No | Progression of disease | Discharge from hospital (best supportive care) |
| Patient 10 | xx | 72 | 1 | Lung | No | C-reactive protein ≥5 mg/dl;  Lactate dehydrogenase ≥240 U/liter | No | None | Hospitalization |
| Patient 11 | xy | 74 | 2 | Bladder | No | Platelet count decrease (G3); | No | Acute respiratory distress syndrome | Death |
| Patient 12 | xy | 73 | 3 | Colon | Yes | C-reactive protein ≥5 mg/dl;  Lactate dehydrogenase ≥240 U/liter | No | Septic shock | Death |
| Patient 13 | xy | 61 | 2 | Lung | No | Lymphocytes decreased (G2);  Lactate dehydrogenase ≥240 U/liter | No | Progression of disease | Death |
| Patient 14 | xx | 84 | 3 | Blood/Bone Marrow | Yes | Platelet count decrease (G3);  C-reactive protein ≥5 mg/dl; | No | Progression of disease | Death |
| Patient 15 | xy | 55 | 2 | Lung | Yes | C-reactive protein ≥5 mg/dl; | No | Progression of disease | Death |
| Patient 16 | xy | 76 | 1 | Lung | Yes | None | No | Progression of disease | Death |
| Patient 17 | xy | 74 | 1 | Lung | No | C-reactive protein ≥5 mg/dl; | Yes (hydroxychloroquine) | Progression of disease | Death |
| Patient 18 | xy | 82 | 2 | Blood/Bone Marrow | No | C-reactive protein ≥5 mg/dl; | Yes (hydroxychloroquine + darunavir/ritonavir) | Acute respiratory distress syndrome | Death |

**Supplementary Table 2**: **characteristics and outcomes of individual patients included in the case series.** The number of each patient corresponds to that reported in figure 1. The grading of abnormal laboratory findings was assigned according to Common Terminology Criteria for Adverse Events v4.0 (CTCAE).
